# Inflammation and Resolution Are Associated with Upregulation of Fatty Acid β-Oxidation in Zymosan-Induced Peritonitis

**DOI:** 10.1371/journal.pone.0066270

**Published:** 2013-06-11

**Authors:** Yusuke Fujieda, Atsushi Manno, Yasuhiro Hayashi, Nelson Rhodes, Lining Guo, Makoto Arita, Takeshi Bamba, Eiichiro Fukusaki

**Affiliations:** 1 Department of Biotechnology, Graduate School of Engineering, Osaka University, Suita, Japan; 2 Asubio Pharma Co., Limited, Kobe, Japan; 3 Metabolon Inc. Durham, North Carolina, United States of America; 4 Department of Health Chemistry, Graduate School of Pharmaceutical Sciences, University of Tokyo, Japan; Friedrich-Alexander-University Erlangen, Germany

## Abstract

Inflammation is a fundamental defensive response to harmful stimuli. However, it can cause damage if it does not subside. To avoid such damage, organisms have developed a mechanism called resolution of inflammation. Here we applied an untargeted metabolomics approach to a sterile and self-resolving animal model of acute inflammation, namely zymosan-induced peritonitis in mice, to examine the effect of inflammation and resolution on the metabolomic profiles. Significant and time-dependent changes in metabolite profiles after zymosan administration were observed in both peritoneal wash fluid (PWF) and plasma. These metabolomic changes correlated well with inflammatory chemokine or cytokine production. In PWF, most of metabolites that could detected increased in zymosan-treated mice, which is suggestive of inflammation, oxidative stress and increased energy demands. In plasma, most metabolites in the central metabolic pathway (glycolysis and TCA cycle) were significantly downregulated after zymosan administration. The concentration of the ketone body 3-hydroxybutyric acid (3-HB) in plasma and PWF increased in zymosan-injected animals indicating upregulation of fatty acid β-oxidation. Increased 3-HB level was observed in the cells that infiltrated into the peritoneal cavity and these infiltrated cells might contribute, at least in part, to the production of 3-HB in the peritoneal cavity.

## Introduction

Inflammation is a fundamental defensive response to harmful stimuli, such as pathogens, damaged cells, or irritants. Inflammation will subside normally if harmful stimuli are removed, but it will cause damage to organisms if inflammation dose not subside, which will eventually lead to chronic inflammation [1]. To avoid this, organisms have developed a mechanism to subside inflammation, namely, resolution of inflammation which is an active process rather than a passive process and mediated by chemical mediators [2].

Among the chemical mediators of resolution are specialized pro-resolving lipid mediators, such as lipoxins, D and E series resolvins, (neuro)protectins, and maresins [3]. Recently, resolvin E3 (17,18-dihydroxyeicosapentaenoic acid) has been added to this category as a novel anti-inflammatory mediator [4].

One of the animal models to study resolution of inflammation is a zymosan-induced peritonitis model in mice. Lower dosage of zymosan causes transient inflammation characterized by neutrophil clearance followed by infiltration of resolution-phase macrophages, while higher dosage of zymosan induces more aggressive and prolonged inflammation [5]. Although transcriptome of resolution-phase macrophages has been analyzed, the gene that are in the process of resolution of inflammation still remain to be fully clarified [6].

Metabolomics is a new approach that involves the determination of changes in the levels of endogenous or exogenous metabolites in biological samples in response to physiological stimuli or genetic modification [7], [8]. The power of metabolomics lies in the global determination of metabolites, or patterns of biomarkers that increase or decrease as the result of a particular disease or injury. In this study, we applied mass spectrometry (MS)-based metabolomic technology to a sterile and self-resolving animal model of acute inflammation and resolution, namely zymosan-induced peritonitis in mice, to examine the effect of inflammation and resolution on the metabolomic profiles.

## Materials and Methods

### Materials and reagents

Zymosan-A was from SIGMA-Aldrich (St. Louis, MO, USA). Isoflurane was from Mylan (Japan) and *N-*Methyl-*N-*trimethylsilyl-trifluoroacetamide (MSTFA) was from GL Science (Japan). Other reagents and materials were of analytical or special grade.

### Preparation of animals

This protocol was approved by the Ethics Committee for Animal Experiments of Asubio Pharma CO., LTD. (Permit Number: AEK-11-175) and carried out in strict accordance with the Guideline for Animal Experiments of the laboratories. Seven-week-old male C57BL/6J mice were purchased from Charles River Japan (Japan). During the acclimation and experimental periods, animals were housed in an air-conditioned animal room (room temperature, 23°C±2°C; relative humidity 50%±20%; 12 h light/dark cycle). The mice were quarantined for 1 week in the animal room assigned for the study and only those without any abnormal findings at the end of this acclimation period were selected for experimentation. CRF-1 pellet diet (Oriental Yeast Co., Ltd., Japan) and tap water were freely available throughout the study. All surgery was performed under inhalational anesthesia with isoflurane, and all efforts were made to minimize suffering.

### Time course of leukocytes cell numbers or populations in peritonitis

To determine the cellular events underlying resolution, we investigated the temporal and differential changes of leukocytes in self-resolving peritonitis exudates from mice using flow cytometry. Peritonitis was induced by an intraperitoneal (i.p.) injection of zymosan-A (1 or 10 mg/mouse) and control mice were injected with vehicle only [9]. At the indicated time points (6 h, 24 h, 48 h, 72 h, 7 days or 9 days), mice were sacrificed, and peritoneal exudates were collected by lavaging with 5 mL of phosphate buffered saline (PBS). The number of animals sacrificed at in each point in each group (10 mg zymosan, 1 mg zymosan, and the control) was 3. Peritoneal exudates without zymosan administration were also collected (0 h). The number of cells that infiltrated the peritoneal cavity was counted by hemocytometry. For determination of cellular composition, peritoneal leukocytes were blocked with anti-mouse CD16/32 blocking antibody (93; eBioscience, San Diego, CA, USA) for 5 min and stained for 20 min with antibodies to either PE-conjugated anti-mouse Gr-1 (RB6–8C5; BD Pharmingen, San Jose, CA, USA), APC-conjugated anti-mouse CD115 (AFS98; eBioscience), PE-conjugated anti-mouse Siglec-F (E50-2440; BD Pharmingen), or FITC-conjugated anti-mouse CCR3 (83101; R&D Systems, Minneapolis, MN, USA). The composition of cell populations was analyzed by fluorescence-activated cell sorting (FACS) using FACSAria (BD Biosciences, San Jose, CA, USA) and the data were analyzed with FACSDiva software (BD Biosciences).

CD115^−^ Gr-1^+^ cells were classified as polymorphonuclear neutrophils (PMNs), CD115^+^Gr-1^−^ were classified as macrophages and CCR3^+^Siglec-F^+^ cells were classified as eosinophils, respectively. Since the number of eosinophils at 0 h (normal) and 6 h after zymosan administration were not counted, the time courses of eosinophils is shown using the data from 24 h, 48 h, 72 h, 7 days and 9 days.

### Metabolomic profiling of peritoneal wash fluid (PWF) and plasma after zymosan-induced peritonitis

Peritonitis was induced by intraperitoneal (i.p.) administration of zymosan-A. Mice were divided into 3 groups: group 1 was administered 1 mg of zymosan, group 2 was administered 10 mg of zymosan, and group 3 was administered vehicle only. Five mice from each group were analyzed at 6, 24, 48, and 72 h after zymosan administration. The number of animals in each group was 20. Mice were deeply anesthetized by isoflurane at the sampling time and blood was collected from heart. Plasma samples were obtained by centrifugation of blood samples at 10,000×*g* for 3 min at 4°C (anticoagulant: heparin sodium; approximately 10 IU/mL plasma). Then a peritoneal lavage was immediately performed with 3 mL of ice-cold PBS, and peritoneal wash fluid (PWF) was collected by centrifugation. PWF and plasma samples were immediately frozen in liquid nitrogen and were stored at −80°C until use.

Cytokines/chemokines concentrations in PWF and plasma were determined using the Bio-Plex mouse cytokine 23-plex assay (catalog no. M60-009RDPD; Bio-Rad, Hercules, CA, USA) according to the manufacturers directions. They were measured with a Luminex Bio-Plex 200 system (Bio-Rad) and then analyzed with Bio-Plex Manager 6.1 software (Bio-Rad). Two plasma samples (zymosan 1 mg, 6 h and 24 h) could not be measured due to shortage of sample volume.

Metabolomic profiling of PWF was performed as described previously [10], [11]. Peritoneal exudate samples were thawed on ice and 300 µL was freeze dried overnight under vacuum, and then dissolved in 100 µL of water and stored at −80°C until extraction. Extraction was performed by adding 450 µL of methanol containing recovery standards and shaking vigorously for 2 minutes. The samples were then centrifuged, the supernatant was removed (MicroLab STAR® robotics; Hamilton, Reno, NV, USA), and then the sample was briefly placed on a TurboVap® (Zymark, Hopkinton, MA, USA) to remove the organic solvent. Each sample was dried under vacuum overnight. The untargeted metabolomic profiling platform employed for this analysis was based on a combination of 3 independent platforms: ultrahigh performance liquid chromatography/tandem mass spectrometry (UHPLC/MS/MS) [10] optimized for basic species, UHPLC/MS/MS optimized for acidic species, and gas chromatography/mass spectrometry (GC/MS) [11]. The LC/MS portion of the platform incorporates a Waters Acquity UPLC system (Waters, Milford, MA, USA) and a Thermo-Finnigan LTQ mass spectrometer (Thermo-Fisher Scientific; Waltham, MA, USA) including an electrospray ionization (ESI) source and linear ion-trap (LIT) mass analyzer. Aliquots of the vacuum-dried samples were reconstituted, one each in acidic or basic LC-compatible solvents containing 8 or more injection standards at fixed concentrations (to ensure both injection and chromatographic consistency). Extracts were loaded onto columns (UPLC BEH C18-2.1×100 mm, 1.7 µm; Waters) and gradient-eluted with water and 95% methanol containing 0.1%formic acid (acidic extracts) or 6.5 mM ammonium bicarbonate (basic extracts). Samples for GC/MS analysis were dried under vacuum desiccation for a minimum of 18 h prior to derivatization under nitrogen using bistrimethyl-silyl-trifluoroacetamide (BSTFA). The GC column was 5% phenyl dimethyl silicone and the temperature ramp was from 60°C to 340°C over a 17-min period. All samples were then analyzed on a Trace DSQ fast-scanning single-quadrupole mass spectrometer (Thermo-Fisher Scientific) using electron ionization. Chromatographic separation was performed followed by full-scan mass spectroscopy to record the retention time and molecular weight (m/z) of all detectable ions present in the samples. Additionally, in the 2 LC methods (which are capable of tandem mass spectrometry), the retention time, molecular weight (m/z), and MS/MS of all detectable ions were recorded. Metabolites were identified by automated comparison of the ion features in the experimental samples to a reference library of chemical standard entries that include retention time, molecular weight (m/z), preferred adducts, and in-source fragments as well as their associated MS/MS spectra. For ions that were not covered by the standards, additional library entries were added based on their unique retention time and ion signatures.

Plasma aqueous metabolites were measured by GC/MS. To extract plasma metabolites, 50 µL of plasma were mixed with 950 µL of solvent mixture (MeOH∶H_2_O∶CHCl_3_ = 2.5∶1∶1 [v/v]) containing 50 µL of 50 µM ribitol (internal standard) dissolved in distilled water. The mixtures were incubated with shaking 1,200 rpm at 37°C for 30 min, and then centrifuged at 10,000×g for 3 min at 4°C. A 900 µL aliquot of the supernatant was transferred to a clean 1.5 mL tube, and 400 µL of distilled water were added. After mixing, the solutions were centrifuged at 10,000× g for 3 min at 4°C, and the supernatant (900 µL) was dispensed into 2 clear tubes. Then, the aqueous phase was collected and evaporated by vacuum centrifugation for 3 h and freeze-dried overnight. For oximation, 70 µL of methoxyamine hydrochloride in pyridine (20 mg/mL) were added and incubated at 30°C for 90 min. For trimethylsilylation, 50 µL of MSTFA were added and incubated at 37°C for 30 min. The mixture was centrifuged, and then a 1 µL aliquot of the resultant supernatant was injected into GC/MS in split mode (15/1, v/v). A GCMS-QP2010 Ultra (Shimadzu, Japan) was used for the analysis and the analytical conditions have been reported previously [12]. Specifically, the column was a 30 m ×0.25 mm i.d. fused silica capillary column coated with 0.25 µm CP-SIL 8 CB low bleed/MS (Agilent Technologies, Santa Clara, CA, USA). The front inlet temperature was set to 230°C. The helium gas flow rate through the column was 1 mL/min. The column temperature was held at 80°C for 2 min isothermally and then raised 15°C/min to 330°C and held there for 6 min isothermally. Twenty scans per second were recorded over the mass range 85 to 500 m/z. Peak detection and identification procedures were the same as previously reported [13]. Briefly, peak detection and alignment were performed using MetAlign software (Wageningen UR, The Netherlands, freely available at http://www.pri.wur.nl/UK/products/MetAlign/) [14]. The resulting data were exported in a CSV-format file. After the CSV-format file was imported using AIoutput software, peak identification and prediction were performed, and then output as an organized data matrix.

### Ketone body formation in the liver and in the cells that infiltrated into the peritoneal cavity after zymosan-induced peritonitis

To determine the cellular source of the ketone bodies, peritonitis was induced by intraperitoneal (i.p.) administration of zymosan-A. Mice were divided into 2 groups: group 1 was administered 10 mg of zymosan and group 2 (control) was administered vehicle. Mice were analyzed 24 and 48 h after zymosan administration. The number of animals in each group was 10, and 5 animals were sampled at each time point. Mice were deeply anesthetized with isoflurane at sampling time, and blood was collected from heart. Plasma samples were obtained by centrifugation (anticoagulant: heparin sodium; approximately 10 IU/mL plasma) as described previously. After blood sampling, peritoneal lavage was obtained with 3 mL of ice-cold PBS and both exudate cells and PWF were collected. Livers were also collected, and the weight of each liver sample was recorded. The number of infiltrated cells was counted by hemocytometry. All samples were immediately frozen in liquid nitrogen and stored at −70°C. Liver samples were homogenized on ice by adding 10 volumes (v/w) of cold PBS before measurement. Infiltrated cells were homogenized by adding 1 mL of cold PBS. Liver and infiltrated cell homogenates were centrifuged at 10,000× g for 3 min at 4°C and the supernatants were used for measurement. The concentration of 3-HB in each sample was quantified using the EnzyChrom ketone body assay kit (catalog no. EKBD-100; BioAssay Systems, Hayward, CA, USA) according to the manufacturer's instructions. 5 µL of infiltrated cell homogenate, 20 µL of liver homogenate, 80 of PWF and 5 µL of plasma were used for the measurement, respectively. The absorbance at 340 nm of each sample was measured by a microplate reader (SPECTRAmax Plus 384; Molecular Devices, Sunnyvale, CA, USA) to calculate the 3-HB concentration.

### Statistical analysis

Dunnett's two-tailed test and Student's t-test were used for statistical analyses and were calculated using JMP 10 software (SAS Institute Inc., Cary, NC, USA). *P* values <.05 were considered statistically significant. Principal component analysis (PCA) were performed using SIMCA-P+ software (Ver. 12.0, Umetrics Inc, Umeå, Sweden).

## Results

### Time course of changes in leukocyte cell numbers during peritonitis

**Figure 1:**
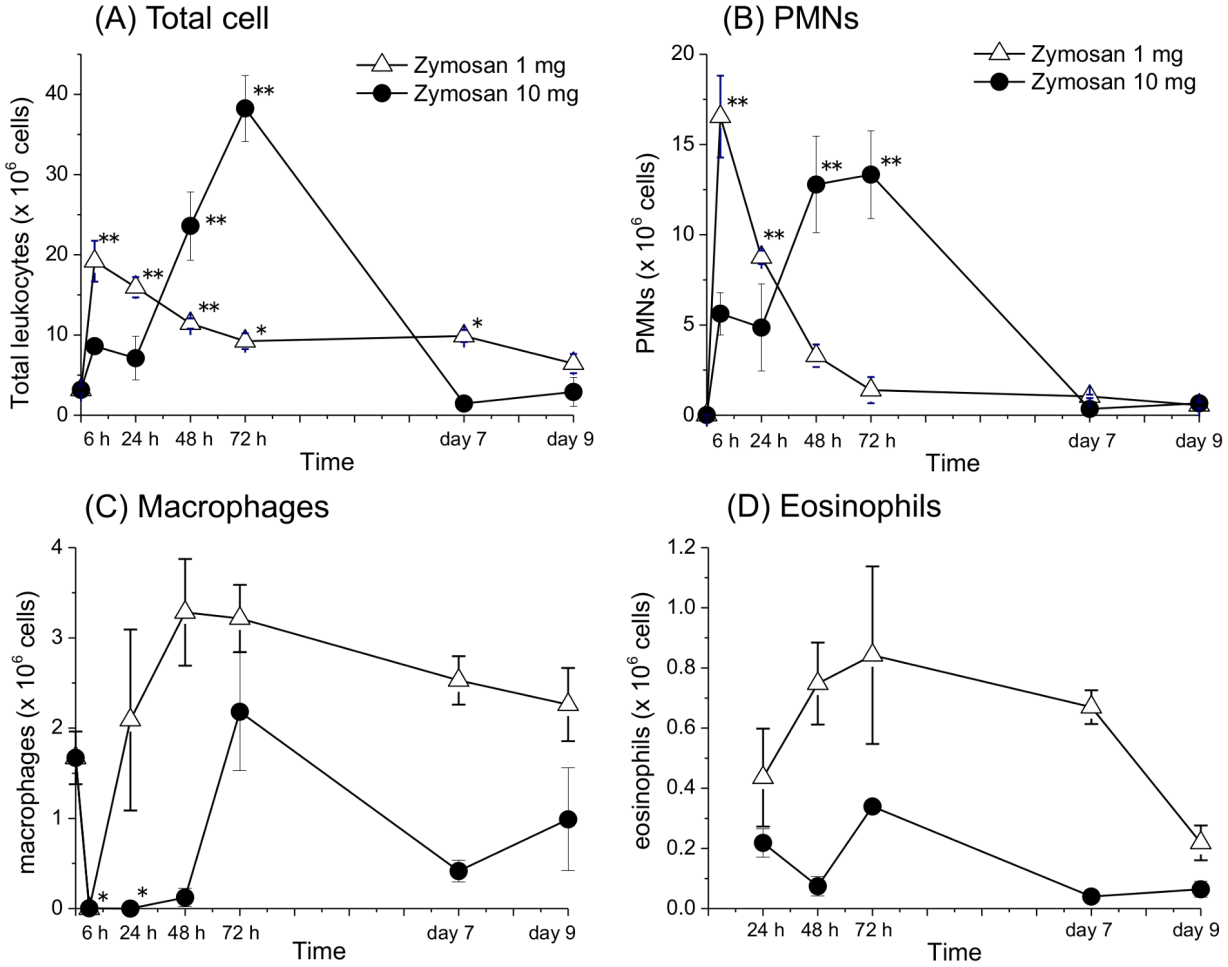
Time courses of leukocytes infiltrated into peritoneal cavity after an intraperitoneal administration of zymosan (1 or 10 mg). (A) Total cell number, (B) cell number of PMNs as determined by CD115^−^ Gr-1^+^ cells, (C) cell number of macrophages as determined by CD115^+^Gr-1^−^ cells, (D) cell number of eosinophils as determined by CCR3^+^Siglec-F^+^ cells. The asterisks indicate significant differences (*P<.05 and **P<.01) compared to the 0 h (control). Since the numbers of eosinophils were not counted at 0 h (normal) or 6 h after administration of zymosan, statistical analysis of eosinophil numbers could not be performed. (n = 3 per group mean±s.e.m.)

After the administration of low dose of zymosan (1 mg), total cell numbers increased with a maximal infiltration at 6 h [19.2±2.6×10[6] cells] (Figure 1A) and the leukocytes were predominantly composed of PMNs, as determined by CD115^−^ Gr-1^+^ cells (Figure 1B). As PMN numbers decreased, CD115^+^Gr-1^−^ cells, which were classified as macrophages, gradually increased and at 48–72 h became the major cell type in the peritoneal exudates (Figure 1C). In addition, eosinophil population characterized by CCR3^+^Siglec-F^+^ also increased (Figure 1D). In contrast, administration of a high dose of zymosan (10 mg) delayed the increase in total cells and PMNs with maximal infiltration at 72 h [38.3±4.1×10[6] cells] (Figure 1A, 1B). The numbers of total cells and PMNs finally decreased to control levels on day 7 or day 9 after zymosan administration, which means the inflammation induced by a high dose of zymosan finally resolved. The numbers of both macrophages and eosinophils were low at 24 and 48 h but increased at 72 h in the group that was administered 10 mg of zymosan. These time courses of leukocytes after the peritonitis are consistent with previous findings [5], [15].

### Time course of cytokines and chemokines concentration in PWF and plasma

**Figure 2:**
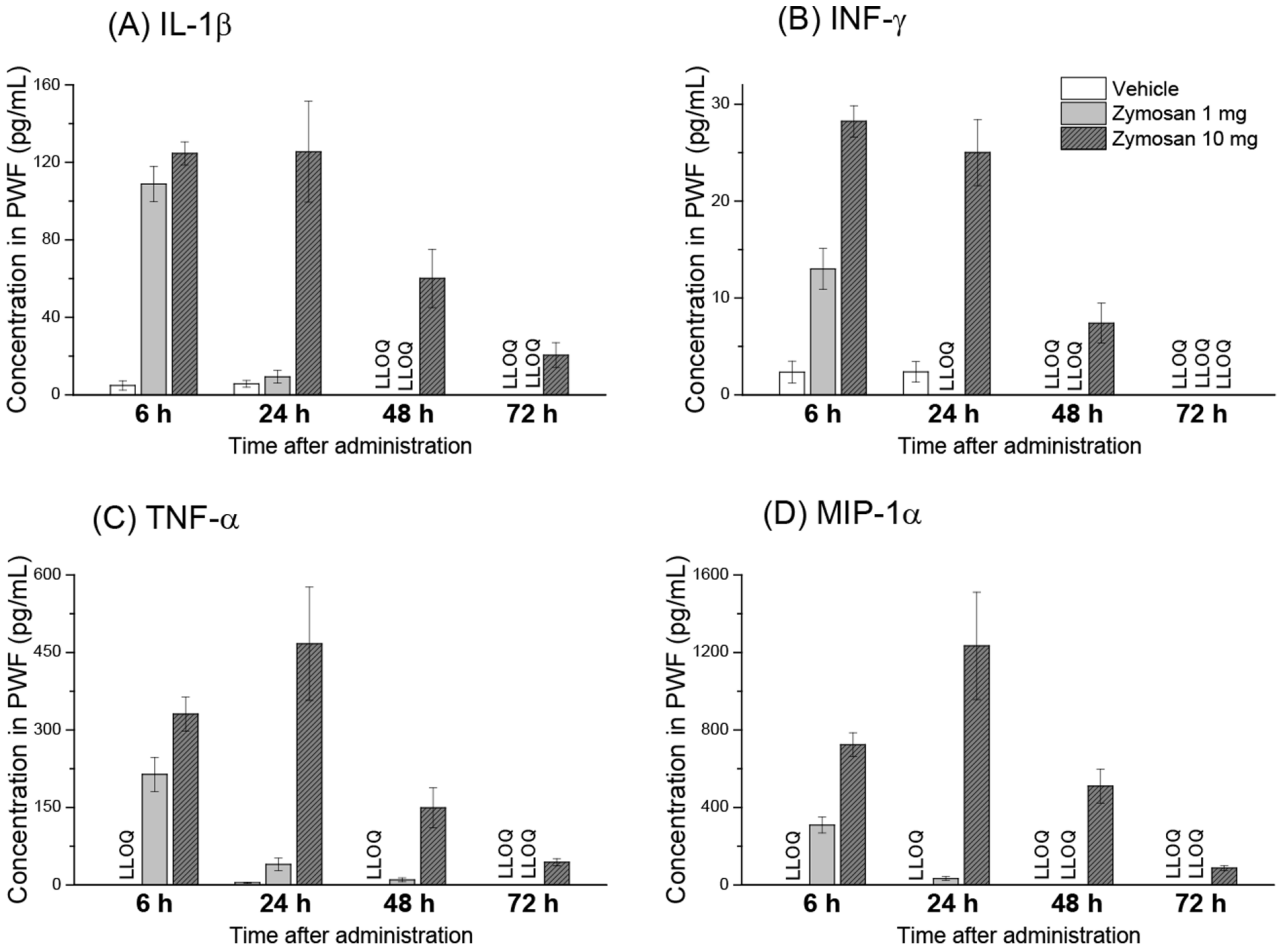
Concentration time courses of IL-1β, TNF-α, IFN-γ and MIP-1α in peritoneal wash fluid (PWF) after an intraperitoneal administration of zymosan or vehicle control in mice. Cytokine and chemokine levels are expressed as mean±s.e.m. (n = 5). LLOQ means lower limit of quantification.

Summary of cytokines and chemokines measurement in PWF and plasma are listed in Dataset S1. Figure 2 shows the time courses of IL-1β, IFN-γ, TNF-α and MIP-1α, which are the typical inflammatory cytokines or chemokines, concentration in PWF. At 6 h, these inflammatory cytokines and chemokines levels in PWF and were higher in both zymosan 1 mg and 10 mg than vehicle control. At 24 h and 48 h, higher levels of these inflammatory cytokines and chemokines concentrations were sustained in zymosan 10 mg mice, but not in zymosan 1 mg mice. These results suggest that inflammation is triggered more severely in zymosan 10 mg group than in zymosan 1 mg group and that resolution of inflammation is delayed in zymosan 10 mg.

### Global metabolomic changes in PWF following zymosan-induced peritonitis

302 metabolites were detected of which 196 matched known structures in our chemical reference library in PWF samples. The metabolites matched with known chemical structures and their statistical comparisons by Dunnett's test are listed in Dataset S2.

**Figure 3:**
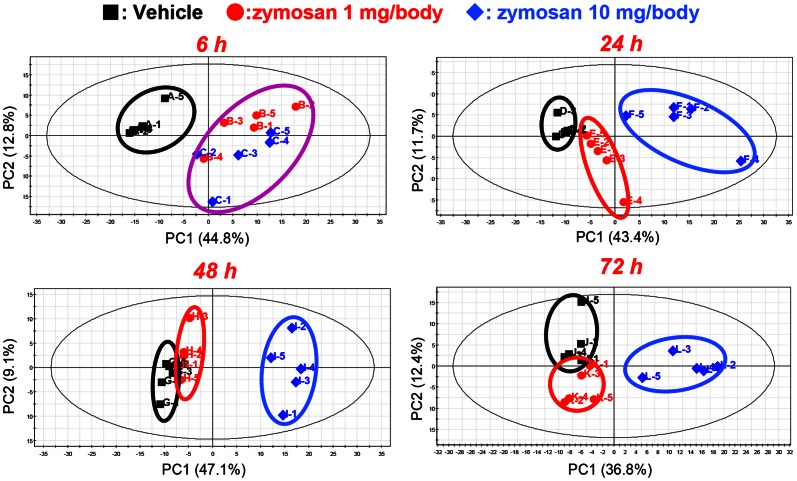
Time course of principle component analysis (PCA) score plot of PWF metabolomics data. Black symbols: control animals, blue: zymosan 1 mg administered animals, red: zymosan 10 mg administered animals, respectively. Black eclipse in the score plot illustrates the 95% confidence regions.

Substantial metabolic changes in PWF occurred during peritonitis, and 6 h after zymosan administration, more than 50% of the profiled metabolites differed significantly compared to vehicle controls. Figure 3 showed PCA score plots of PWF metabolites at each time point. Clear metabolic separation between the sham and peritonitis animals was observed in PCA score plot at 6 h after the induction of peritonitis. Metabolomic profiles of animals administered with 1 mg of zymosan showed that there were reduced biochemical changes relative to the vehicle control from 48 h. This means that the metabolomic profiles of the 1 mg group were recovered by the 48 h time point. On the other hand, metabolomic profile of animals administered with 10 mg of zymosan were clearly different from that of control animals during the observation period in this study; even at 72 h after zymosan administration.

In the early phase (6 h), most metabolites that were detected by our platforms were higher in zymosan-administered mice than in vehicle-administered control mice. Observations consistent with known effects of zymosan administration [9], including significant increases in inflammatory compounds, such as polyunsaturated fatty acids (PUFAs) and kynurenine, as well as increased lysophospholipids levels were identified (Table 1). In addition, increased glutathione synthesis and turnover, changes in purine catabolism were observed in zymosan administered mice (Table 1). Most of these changes observed in the 1 mg zymosan-administered animals returned to control levels at the 48 and 72 h time points. Biochemical changes indicative of inflammation were still present in animals administered with 10 mg of zymosan after 72 h.

**Table 1:** Summary of altered metabolites in peritoneal wash fluid (PWF) after zymosan intraperitoneal (i.p.) administration.

	Zymosan 1 mg vs. Vehicle				Zymosan 10 mg vs. Vehicle			
Metabolite	Fold change				Fold change			
*Polyunsaturated fatty acids (PUFAs)*	**6 h**	**24 h**	**48 h**	**72 h**	**6 h**	**24 h**	**48 h**	**72 h**
Arachidonate (20∶4n6)	**7.85**	**2.44**	**1.78**	**1.50**	**10.35**	**6.32**	**4.39**	**2.70**
Docosahexaenoate (DHA; 22∶6n3)	**16.84**	2.55	1.79	1.93	**23.05**	**11.16**	**7.49**	**4.68**
Docosapentaenoate (DPA; 22∶5n3)	**4.38**	1.73	1.15	1.53	**5.14**	**4.68**	**3.03**	**2.15**
Eicosapentaenoate (EPA; 20∶5n3)	**2.71**	1.25	1.10	0.92	**3.44**	**2.56**	**2.55**	1.34
*Lysolipids*								
1-Arachidonoyl-GPC (20∶4)	**6.21**	1.64	**2.56**	**2.18**	**6.66**	**2.51**	1.77	1.14
1-Docosahexaenoyl-GPC (22∶6)	4.75	2.77	1.62	1.41	**6.19**	**4.89**	2.17	1.55
1-Oleoyl-GPC (18∶1)	4.39	2.27	3.91	0.85	**5.47**	**7.69**	3.81	1.09
1-Oleoyl-GPE (18∶1)	**3.54**	1.94	1.26	1.25	2.48	**3.42**	**3.64**	**1.89**
1-Palmitoyl-GPC (16∶0)	3.31	2.96	5.52	1.08	4.15	**12.93**	**11.32**	1.92
1-Stearoyl-GPC (18∶0)	4.36	6.22	5.32	1.56	5.56	**20.95**	**22.26**	3.91
*Tryptophan metabolism*								
Tryptophan	**2.88**	**2.07**	1.43	1.00	**3.19**	**3.35**	**2.79**	**2.23**
Kynurenine	**2.41**	1.78	0.83	1.24	**2.34**	**4.47**	**3.87**	2.18
Nicotinamide	**2.03**	1.28	1.16	0.74	**2.06**	0.68	**1.66**	1.25
*Glutathione Synthesis and Turnover*								
Glutathione, oxidized (GSSG)	**4.60**	**1.79**	0.90	0.66	**2.63**	**1.74**	1.42	0.93
Cysteine-glutathione disulfide	**2.85**	1.99	1.05	0.92	**2.16**	**2.63**	**2.18**	**1.75**
Cysteine	1.25	1.03	1.27	1.21	2.07	**2.52**	**3.65**	1.68
5-Oxoproline	**2.93**	**1.79**	1.25	1.06	**2.95**	**2.36**	**3.31**	1.37
2-Aminobutyrate	**1.86**	0.82	1.07	1.06	**1.78**	1.43	**2.68**	1.44
*Purine Catabolism*								
AMP	**3.68**	**3.85**	**2.89**	1.94	0.48	0.29	1.25	**2.77**
Adenosine	**0.18**	**0.15**	**0.15**	**0.19**	**0.12**	**0.06**	**0.14**	**0.09**
Inosine	0.66	**0.32**	1.08	**0.29**	0.65	**0.28**	0.75	**0.24**
Urate	**3.23**	1.29	1.28	0.66	**2.74**	**2.24**	**3.54**	1.36
*Carnitines*								
2-Methylbutyroylcarnitine (C5)	**1.96**	1.29	1.03	0.97	**2.18**	**1.69**	1.34	1.28
3-Dehydrocarnitine	**2.03**	1.38	1.10	0.94	**1.82**	**1.55**	**2.07**	**1.59**
3-Methylglutaroylcarnitine (C6)	**3.73**	1.72	1.49	0.74	**3.56**	**6.36**	**9.55**	1.01
Acetylcarnitine (C2)	**2.60**	1.46	0.92	**0.78**	**2.44**	**1.88**	**2.37**	**1.25**
Butyrylcarnitine (C4)	**3.34**	**1.40**	0.99	1.07	**2.30**	1.29	1.31	1.11
Carnitine	**1.65**	**1.19**	1.06	0.97	1.22	**0.80**	**1.48**	**1.46**
Deoxycarnitine	**1.69**	**1.72**	1.09	0.75	**1.99**	1.19	**1.39**	**1.84**
Hexanoylcarnitine (C6)	**2.47**	1.43	0.98	1.29	**3.01**	**2.19**	**3.10**	1.48
Isobutyrylcarnitine (C4)	**1.76**	1.70	1.05	0.71	**1.89**	**1.98**	**1.65**	**1.35**
Isovalerylcarnitine (C5)	1.65	1.48	1.12	0.94	**2.10**	**3.05**	**2.96**	**2.01**
Propionylcarnitine (C3)	**1.73**	**1.45**	1.25	0.83	1.56	1.01	**1.65**	**1.86**

Statistically significant changes are in bold (*P*<0.05, compared with vehicle control). GPC refers to glycerophosphocholine and GPE refers to glycerophosphoethanolamine.

The time course of changes in adenosine and adenosine monophosphate (AMP) concentrations in PWF were distinct from those of other metabolites (Figure S1). AMP levels in the 1 mg and 10 mg zymosan-treated groups were clearly different. The AMP concentrations in the 1 mg group at 6 to 48 h after administration were much higher than those of the 10 mg group, whereas at 72 h, the AMP concentration of the 10 mg group was higher than that of the 1 mg group. In contrast, the concentration of adenosine, which is a neighbor metabolite of AMP in the metabolic pathway, in PWF decreased significantly at 6 through 72 h in both the 1 mg and 10 mg zymosan-treated groups.

**Figure 4:**
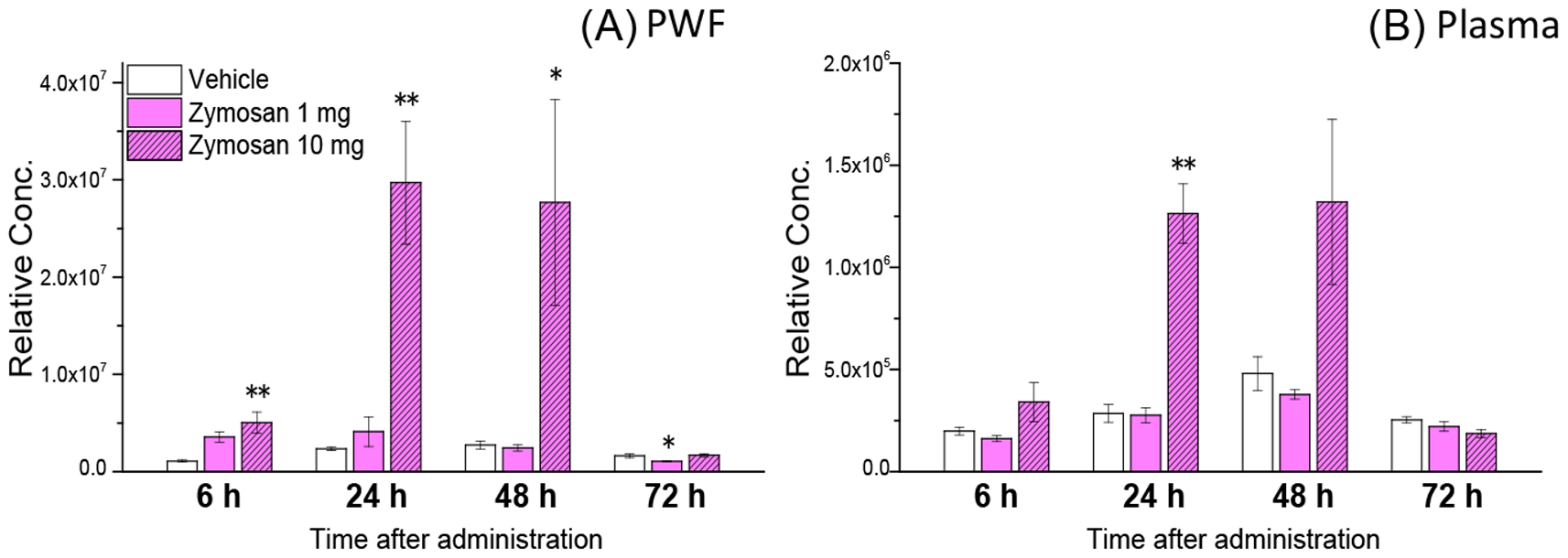
Relative concentration time courses of 3-hydroxybutyric acid in PWF (A) and plasma (B) after an intraperitoneal administration of zymosan or vehicle control in mice. These metabolites levels are expressed as mean±s.e.m (n = 5). *P<0.05, **P<0.01, compared with vehicle control.

Significant increases in the levels of the ketone body, 3-hydroxybutyric acid (3-HB), (Figure 4A), as well as the short-chain acylcarnitine, acetylcarnitine were observed in zymosan high dosed treated mice (Table 1). Since ketone bodies are produced as by-products of fatty acids β-oxidation for energy in the liver [16], [17], these changes are suggestive of increased fatty acid mobilization and β-oxidation.

### Metabolomic changes in plasma following zymosan-induced peritonitis

99 metabolites were detected of which 76 matched known structures in our chemical reference library in PWF samples. The metabolites matched with known chemical structures and their statistical comparisons by Dunnett's test are listed in Dataset S3.

Peritonitis induced dramatic metabolic changes in plasma, and more than 40% of the profiled metabolites that could be detected were significantly altered 6 h after zymosan administration. PCA score plots of plasma metabolites at each time point are presented in Figure S2. Similar to the case of PWF, plasma metabolomic profile of animals administered with 1 mg of zymosan recovered by the 48 h and there were reduced biochemical changes relative to the vehicle control. On the other hand, the metabolomic profiles of animals administered with 10 mg of zymosan were clearly different from that of control during the observation period in this study.

Remarkable changes in energy related metabolites were observed in plasma. Table 2 shows a summary of the time courses of plasma metabolites in the central metabolic pathway (glycolysis and TCA cycle). Most of metabolites in central metabolic pathway including glucose were downregulated during the period of inflammation. Although this downregulation recovered at the 48 and 72 h time points in the 1 mg administered animals, these changes were still present in animals administered with 10 mg of zymosan after 72 h.

**Table 2:** Summary of plasma metabolites in central metabolic pathway (glycolysis and TCA cycle) after zymosan i.p. administration.

	Zymosan 1 mg vs. Vehicle				Zymosan 10 mg vs. Vehicle			
Metabolite	Fold change				Fold change			
*Glycolysis*	**6 h**	**24 h**	**48 h**	**72 h**	**6 h**	**24 h**	**48 h**	**72 h**
Glucose	**0.80**	**0.76**	0.93	0.95	**0.56**	**0.29**	**0.61**	**0.53**
Pyruvate + Oxalacetate	0.97	**0.75**	1.03	0.90	**0.73**	**0.45**	0.71	**0.52**
Lactate	**0.82**	0.91	0.98	0.96	**0.65**	**0.74**	0.80	0.91
*TCA cycle*								
Citrate + Isocitrate	**0.71**	**0.65**	0.96	**0.84**	**0.72**	**0.30**	**0.19**	**0.39**
Alpha-ketoglutarate	**0.44**	**0.22**	**0.59**	**0.33**	**0.32**	**0.19**	**0.08**	**0.09**
Fumarate	**0.44**	**0.38**	1.04	**0.67**	**0.40**	**0.43**	**0.24**	**0.32**
Malate	**0.42**	**0.37**	0.98	0.70	**0.36**	**0.39**	**0.20**	**0.28**

Statistically significant changes are in bold (*P*<0.05, compared with vehicle control).

Significant increases in the plasma levels of 3-HB were also observed in high dose zymosan-treated mice (Figure 4B). Downregulation of metabolites in this central metabolic pathway and the increase of 3-HB are suggestive of increased energy consumption and increased fatty acid β-oxidation during the inflammation caused by the zymosan peritonitis.

### Ketone body concentration in the liver and in the cells that infiltrated into the peritoneal cavity after zymosan-induced peritonitis

The concentrations of 3-HB in the cells that infiltrated into the peritoneal cavity, PWF and plasma in control and zymosan-treated (10 mg) animals are presented in Table 3. The concentrations of 3-HB in liver were not detectable (detection limit; approximately 0.1 µmol/g tissue). Increased levels of 3-HB in PWF and plasma were observed at 24 h and 48 h after induction of peritonitis, which suggests increased fatty acid mobilization and β-oxidation. The increases observed in the total number of cells that infiltrated into the peritoneal cavity after peritonitis were reproduced in this experiment (Table 3). The production of 3-HB in cells that infiltrated into the peritoneal cavity of zymosan-treated animals was significantly higher than that of vehicle control animals not only in total, but also per cell (Table 3).

**Table 3.** Concentrations of 3-HB in the cells that infiltrated into the peritoneal cavity, PWF, and plasma in control and zymosan-treated (10 mg) animals.

**Table:** Table 3. Concentrations of 3-HB in the cells that infiltrated into the peritoneal cavity, PWF, and plasma in control and zymosan-treated (10 mg) animals.

Infiltrated cells into peritoneal cavity	Time	Vehicle	Zymosam (10 mg)
Total cell number (1×10^6^)	24 h	2.24±0.25	6.37±0.72 **
	48 h	2.28±0.28	14.21±5.02 *
3-hydroxybutyric acid (nmol/total cells)	24 h	0.49±0.08	4.74±0.59 **
	48 h	0.29±0.06	9.15±1.27 **
3-hydroxybutyric acid (nmol/1×10^6^ cells)	24 h	0.22±0.03	0.79±0.14 **
	48 h	0.15±0.06	1.06±0.33 *
Liver			
	Time	Vehicle	Zymosam (10 mg)
3-hydroxybutyric acid (nmol/g tissue)	24 h	84.1±17.0	347.8±28.0 **
	48 h	70.2±15.8	1266.1±482.1 *
PWF			
	Time	Vehicle	Zymosam (10 mg)
3-hydroxybutyric acid (nM)	24 h	ND	0.08±0.05
	48 h	ND	0.30±0.09
Plasma			
	Time	Vehicle	Zymosam (10 mg)
3-hydroxybutyric acid (nM)	24 h	0.55±0.07	1.16±0.13 **
	48 h	0.29±0.07	1.63±0.44 *

Concentrations are expressed as mean ± s.e.m (n = 5). The asterisks indicate significant differences (** *P*<.01 and * *P*<.05) compared to the vehicle control. Since the 3-HB concentrations of PWF in vehicle control animals were not detectable (ND, detection limit; 0.001 mM), statistical analysis could not be performed.

## Discussion

The initiation phase of acute inflammation is characterized by the rapid infiltration of PMNs followed by edema formation in response to injury. In the resolution phase, PMNs undergo apoptosis and are ingested by macrophages that emigrate rapidly from the inflamed site to the draining lymph nodes [18]. The time course of leukocytes after peritonitis (Figure 1) shows that zymosan-induced peritoneal inflammation resolved between 24 and 48 h in the low dose zymosan (1 mg) group, whereas inflammation did not resolve even at 72 h in the high dose zymosan (10 mg) group. However, the number of total cells and PMNs finally decreased to control levels on day 7 or day 9 after peritonitis induction, which means that the inflammation induced by high dose zymosan also finally resolved.

In this study we applied biochemical profiling to assess the global metabolic changes associated with zymosan-induced peritonitis in mice. Although some studies have applied metabolomics approach to zymosan peritonitis [15], [19], most of these studies mainly measured lipid mediators to identify the anti-inflammatory substances or to find the mechanism of resolution. This is, to our knowledge, the first study investigating features of aqueous metabolites after zymosan peritonitis. Significant metabolic changes in both PWF and plasma were observed as a result of peritonitis. These changes were predominantly reflective of an inflammatory response because time courses of metabolic features correlated closely to those of chemokines or cytokines level such as IL-1β or TNF-α (Figure 2). In both PWF and plasma, the metabolomic profiles of animals administered with 1 mg of zymosan recovered by 48 h, whereas those of animals administered with 10 mg of zymosan did not recover even at 72 h after administration.

In PWF, most of metabolites that could be detected significantly increased 6 h after zymosan administration. The local inflammatory response is characterized by a sequential release of mediators and the recruitment of different types of leukocytes that become activated at the inflamed site [20]. After i.p. injection of zymosan, total leukocyte numbers increased with a maximal infiltration at the early phase of inflammation [15]. Increases of various metabolites in PWF might be caused by the leakage of plasma into the peritoneal cavity at the onset of inflammation [21]. Among the metabolites in PWF, elevations of PUFAs such as arachidonic acid, EPA or DHA were remarkable at 6 and 24 h in both the 1 mg and 10 mg administered mice as reported previously [22]. Since these PUFAs are the precursors of inflammatory or anti-inflammatory mediators, elevations in PUFAs after the peritonitis might have important roles in the activation or resolution of inflammation. In addition to PUFAs elevation, increased levels of sn1-linked lysophospholipids were observed in PWF after zymosan administration (Table 1). Cell stimulation by various agonists, such as zymosan, leads to the activation of intracellular phospholipases, including phospholipases A2, C, and D [23]. Phospholipase A2 (PLA2) hydrolyzes the fatty acyl group from the sn-2 position of the phospholipid producing a sn-1-linked lysophospholipid. Precursors of pro- or anti-inflammatory mediators such as arachidonic acid, EPA and DHA are linked through sn-2 esterification to phospholipids, thus the control mechanism of PLA2 activity is likely to be involved in the regulation of inflammatory responses.

As shown in Table 1 and Figure S1, the time courses of adenosine and AMP concentrations in PWF were distinctive from those of other metabolites. In the extracellular space, ATP and ADP are rapidly metabolized to AMP by ectonucleoside triphosphate diphosphohydrolase 1 (CD39), and then extracellular AMP is converted to adenosine by ecto-5′-nucleotidase (CD73) [24]. Adenosine has a generally suppressive effect on the activation of immune cells and signals via the G protein-coupled receptors (e.g., A(2A) and A(2B) receptors) that regulate a wide array of physiologic systems and immune homeostasis [25], [26]. These receptors are expressed on a variety of immune cells, and adenosine receptor induction is responsible for gradually dissipating inflammatory responses. Macrophages that engulf apoptotic cells release adenosine, which triggers A(2A) receptors and dampens the inflammatory response during resolution. In this study, we found that the concentration of adenosine in PWF decreased significantly at 6 to 72 h in both the 1 mg and 10 mg zymosan-treated groups. However, the levels of AMP, the precursor of adenosine, in the 1 mg and 10 mg zymosan-treated groups clearly differed. Since the AMP concentration in PWF seemed to increase during the resolution phase, AMP production might play a role in attenuating the inflammatory response. Further studies are needed to evaluate the contribution of purinergic signaling in inflammation and resolution.

Plasma metabolite profiles were also changed significantly during the peritonitis, but these changes were different from those of PWF. One of the most characteristic responses was the decreased levels of metabolites in the central metabolic pathway (glycolysis and TCA cycle) including glucose (Table 2). Although *Shiomi et al*. reported that the serum levels of metabolites in TCA cycle decreased significantly after the induction of dextran sulfate sodium (DSS) induced colitis in mice [27], this is, to our knowledge, the first report that shows the metabolites in the central metabolic pathway decrease significantly in such a short time after the induction of acute inflammation. Considering the upregulation of ketone body; 3-HB in PWF and plasma after mentioned, these changes might mean the energy shortage following the increase of energy consumption in body during the inflammatory phase.

In both PWF and plasma, significant increases in the levels of 3-HB were observed commonly in 10 mg of zymosan administered mice (Figure 3). 3-HB is a ketone body which is produced mainly from the β-oxidation of fatty acids, and is exported to peripheral tissues for use as an energy source. The term ketone body refers to 3 molecules, acetoacetate, 3-HB, and acetone. 3-HB and acetoacetate transport energy from the liver to the other tissues and acetone is generated by spontaneous decarboxylation of acetoacetate [28]. Moreover, remarkable increases of concentration in PWF of short-chain acylcarnitine, acetylcarnitine were also observed (Table 1). Upregulation in the levels of 3-HB and acylcarnitines have been associated closely with increased β-oxidation. Recently, *Liu et al*. showed a switch of energy source from glucose to fatty acid oxidation during the acute inflammatory response in mice sepsis model [29]. There are several reports of ketoacidosis occurring as a complication of influenza infection in humans [30], [31]. Our metabolomics results firstly demonstrated the upregulation of fatty acid β-oxidation in metabolite level that might be caused by the increase of energy consumption during acute inflammation. *Stables et al*. reported comprehensive transcriptomic analyses of murine resolution-phase macrophages in zymosan-induced peritonitis [6]. Their results show that the expression of some genes related to fatty acid oxidation (e.g. Fabp3, Acly) and the central metabolic pathway were significantly altered. These transcriptomic data are consistent with our results and support the upregulation of fatty acid β-oxidation in zymosan peritonitis.

To determine the cellular source of 3-HB, an additional experiment was conducted. In this experiment, increased levels of 3-HB were also observed in PWF and plasma, and upregulation of 3-HB production was also observed after peritonitis was induced with a high dose of zymosan in the cells that infiltrated into the peritoneal cavity (Table 3). The concentrations of 3-HB in liver (detection limit; approximately 0.1 µmol/g tissue) were not detectable. Although liver is one of the major organs that undergoes fatty acid β-oxidation and produces ketone bodies, 3-HB produced in the liver might transfer out of cells rapidly by transporters such as monocarboxylate transporter (MCT)-1 [32]. Most of the 3-HB in circulating plasma and in the body seems to be produced in the liver or other organs. However, in the peritoneal cavity, it is possible that infiltrated cells contributed, at least in part, to the increase in 3-HB. Which types of cells produce 3-HB in the peritoneal cavity? In the resolution phase, not only myeloid cells but lymphoid cells are attracted to the peritoneal cavity [5] and lymphoid cells are known to use β-oxidation as their main energy-producing pathway upon activation [33]. However, since these lymphoid cells represent only a small portion of the total cells in the PWF [5], this does not seem to be very likely. Because most of the infiltrated cells are myeloid cells [5], it is possible that these myeloid cells may exhibit enhanced β-oxidation, which changes the metabolic profile.

Does the upregulation of fatty acid β-oxidation promote resolution? It is well known that peroxisome proliferator-activated receptor alpha (PPARα) agonists such as ureido-fibrate-5 and GW7647 stimulate the fatty acid β-oxidation in the liver and skeletal muscle [34], [35]. Many studies have demonstrated that PPARα mediates the anti-inflammatory effect [36] in mice with DSS-induced colitis or acute ischemia/reperfusion [37], [38]. Although PPARα ligands reduce the levels of IL-1, TNF-α and inducible nitric oxide synthase (iNOS) by inhibiting translocation of the p65 subunit of nuclear factor κ-B (NF-κB) [39], the precise mechanisms underlying the anti-inflammatory effects of PPARα remain unknown. One possible mechanism is that it provides a sufficient energy supply during the inflammation. Our metabolomics results indicated that a significant increase in energy consumption and upregulation of fatty acid β-oxidation occurs during inflammation after zymosan-induced peritonitis. If we could enhance the fatty acid β-oxidation and supply sufficient energy during the inflammation, the resolution of inflammation might be promoted. Another possible mechanism is 3-HB-mediated activation of GPR109A. A G-protein-coupled receptor (GPR109A/HM74A) was found to mediate the antilipolytic effect of niacin [40], and 3-HB is the endogenous ligand of this receptor [41]. Recently, some researchers have reported the anti-inflammatory effect of GPR109A agonists. *Gambhir et al*. investigated the potential anti-inflammatory action of 3-HB in cultured human and mouse retinal pigment epithelial cells and demonstrated that 3-HB potently suppressed TNF-α-induced expression and release of the proinflammatory cytokine IL-6 and the chemokine Ccl2 by GPR109A-dependent mechanisms [42]. *Digby et al*. also demonstrated that the anti-inflammatory effects of nicotinic acid, a GPR109A ligand, in human monocytes were mediated by GPR109A [43]. 3-HB production in the cells that infiltrated into the peritoneal cavity was comparably high after 10 mg zymosan administration; therefore, it is possible that the increased 3-HB level contributed to the resolution process in this model. Upregulation of fatty acid β-oxidation might be an important process for promoting resolution and treating inflammatory diseases.

## Supporting Information

Dataset S1 **Summary of cytokines and chemokines measurement in peritoneal wash fluid (PWF) and plasma after zymosan-induced peritonitis (n = 5, MEAN).** LLOQ and ULOQ mean lower limits of quantitation and upper limits of quantitation, respectively.
(XLSX)

Dataset S2 **Summary of metabolites in PWF with known chemical structures that show time courses of relative concentration after zymosan-induced peritonitis (n = 5, MEAN).** The values in bold font are statistically significant (*P*<.05) by Dunnette's method vs. vehicle group.
(XLSX)

Dataset S3 **Summary of metabolites in plasma with known chemical structures that show time courses of relative concentration after zymosan-induced peritonitis (n = 5, MEAN).** The values in bold font are statistically significant (*P*<.05) by Dunnette's method vs. vehicle group.
(XLSX)

Figure S1 **Time courses of the relative concentrations of adenosine and AMP in PWF after intraperitoneal administration of zymosan or vehicle control in mice.** Metabolite levels are expressed as mean ± s.e.m (n = 5). The asterisks indicate significant differences (*P<.05 and **P<.01) compared to the vehicle control.
(PDF)

Figure S2 **Time course of PCA score plot of plasma metabolomics data.** Black symbols: control animals, blue: zymosan 1 mg treated animals, red: zymosan 10 mg treated animals, respectively. Black eclipse in the score plot illustrates the 95% confidence regions.
(PDF)
