# Supplementary figures and images for: Inflammation and Resolution Are Associated with Upregulation of Fatty Acid β-Oxidation in Zymosan-Induced Peritonitis
Source: PLoS One. 2013 Jun 11;8(6):e66270. doi: 10.1371/journal.pone.0066270 (PMC3679047; doi:10.1371/journal.pone.0066270)

## adenosine

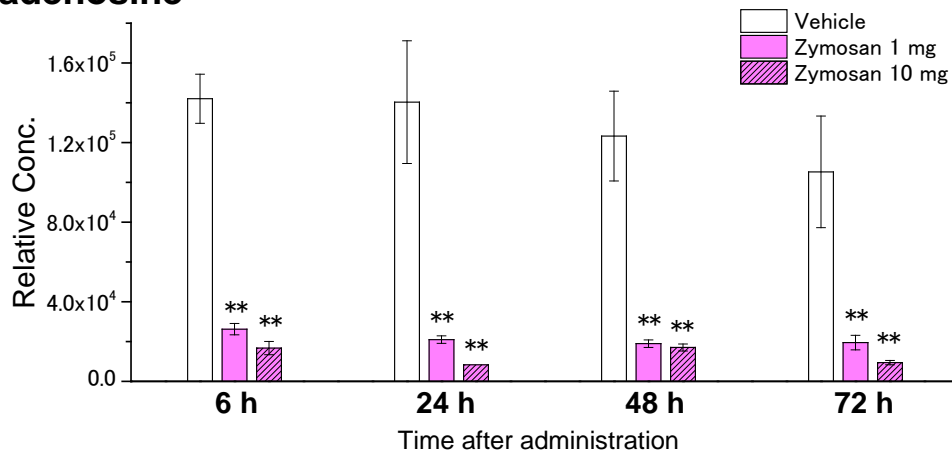

## AMP

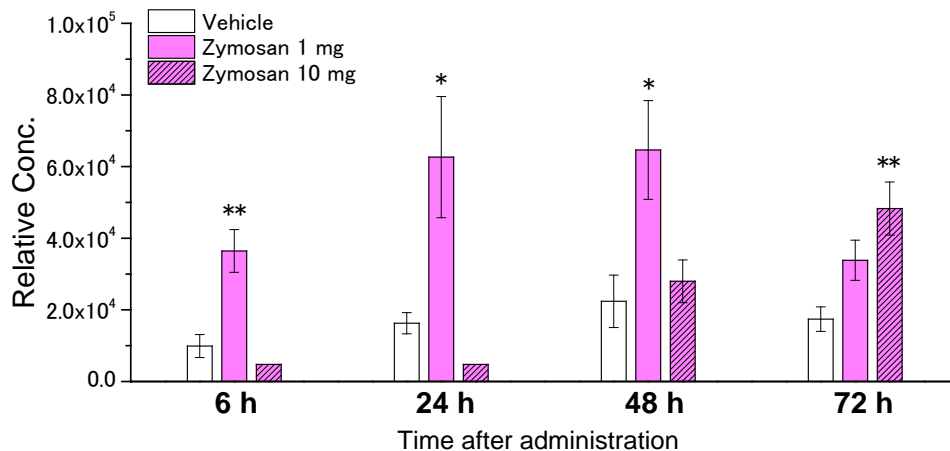

Supplement: Figure S1 — Time courses of the relative concentrations of adenosine and AMP in PWF after intraperitoneal administration of zymosan or vehicle control in mice. Metabolite levels are expressed as mean ± s.e.m (n = 5). The asterisks indicate significant differences (*P<.05 and **P<.01) compared to the vehicle control. (PDF) [file pone.0066270.s004.pdf]
