# Supplementary material for: Inflammation and Resolution Are Associated with Upregulation of Fatty Acid β-Oxidation in Zymosan-Induced Peritonitis
Source: PLoS One. 2013 Jun 11;8(6):e66270. doi: 10.1371/journal.pone.0066270 (PMC3679047; doi:10.1371/journal.pone.0066270)
Supplement: Figure S2 — Time course of PCA score plot of plasma metabolomics data. Black symbols: control animals, blue: zymosan 1 mg treated animals, red: zymosan 10 mg treated animals, respectively. Black eclipse in the score plot illustrates the 95% confidence regions. (PDF) [file pone.0066270.s005.pdf]

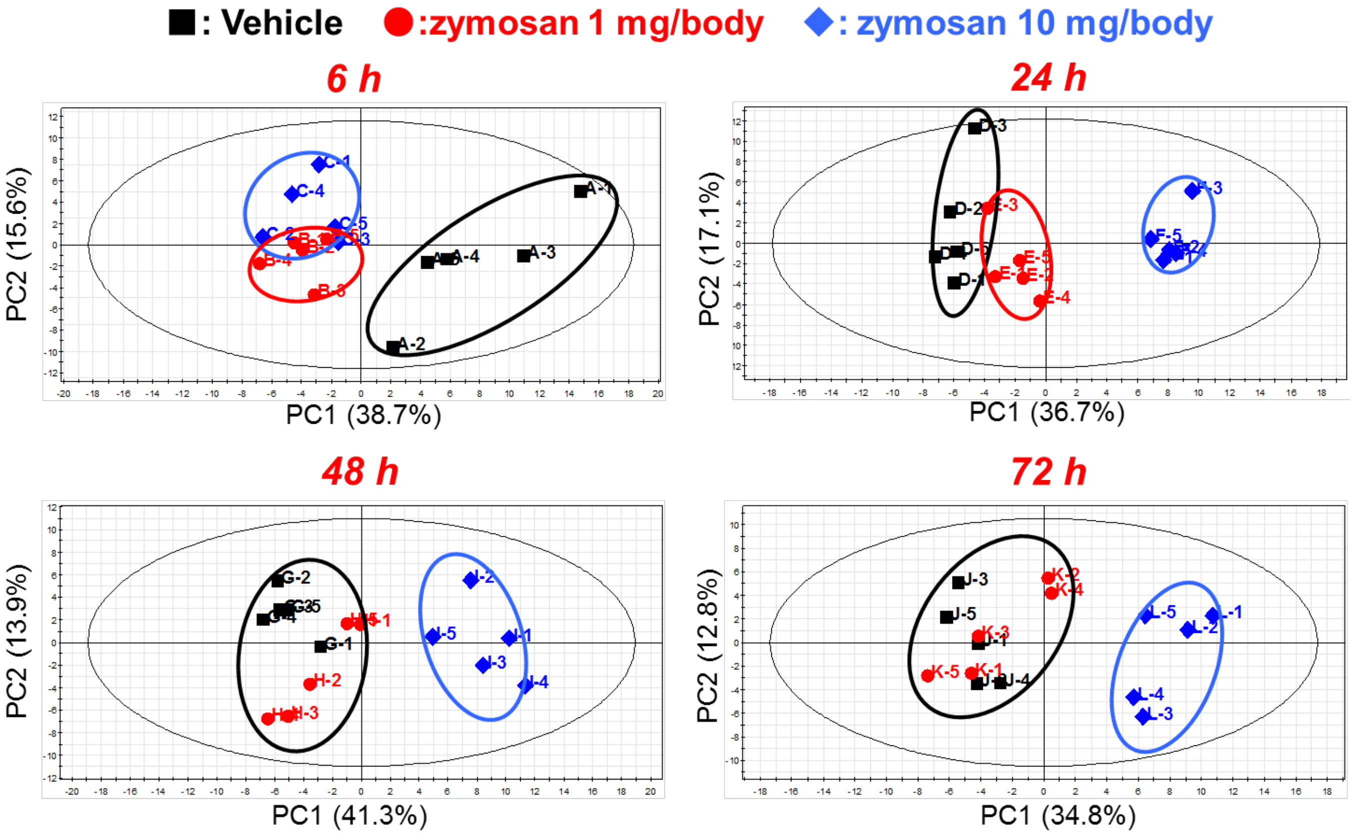

Figure S1

Time course of principle PCA score plot of plasma metabolomics data. Black symbols: control animals, blue: zymosan 1 mg treated animals, red: zymosan 10 mg treated animals, respectively. (PDF)
